# Supplementary material for: A Centrifuge-Based Method for Identifying Novel Genetic Traits That Affect Root-Substrate Adhesion in Arabidopsis thaliana
Source: Front Plant Sci. 2021 Feb 23;12:602486. doi: 10.3389/fpls.2021.602486 (PMC7959780; doi:10.3389/fpls.2021.602486)
Supplement: Supplementary file 1 [file Data_Sheet_1.docx]

***Supplementary Material***

**Table S1** Centrifuge assay troubleshooting

| ***Problem observed*** | ***Possible reason(s)*** | ***Possible solution(s)*** |
| --- | --- | --- |
| Candidate lines and wild-type seedlings significantly differ in size (e.g. in cotyledon size and root length). | Differences in germination rates and development rates of particular lines (e.g. due to age of seed stocks). It is important for candidate lines and wild-type plants to be comparable in size and at the same developmental stage for comparisons of gel-adhesion to be valid. | 1. Set up a time-lapse experiment to compare the developmental stage of candidate line(s) to wild-type seedlings. 2. Decrease or increase the stratification periods of particular lines. 3. Extend the growing period of particular lines (by e.g. +12 hrs for lines developing slowly or -12 hrs for lines developing quickly). 4. Generate new seed stocks. |
| The seedlings are growing into the gel. | The gel has been pierced when the seed was sown or the angle at which the Petri plates have been orientated during the growing period was above or below ~80º causing the roots to grow into the gel. | Regularly check that seedlings grow down the surface of the gel and correct the plate angle if necessary. |
| The gel is shattering during centrifugation | Possible causes include:   1. The medium was too hot when poured so water evaporated, resulting in a higher than intended and/or uneven gel strength. 2. The medium was reheated prior to pouring which changed the gel composition. 3. The surface of the gel was pierced during seed planting, initiating local gel fractures. 4. The Petri plates have been roughly handled, causing fractures. 5. The acceleration and/or deceleration speed on the centrifuge was too high. | 1. Pour the medium into the Petri plates when the media has cooled significantly (and is not steaming). Ensure the medium is thoroughly mixed and bubble free when poured. 2. Do not reheat medium. Store in an oven at 50-60ºC to prevent solidification prior to pouring. 3. Use a desk lamp and magnifier when sowing plates to ensure you do not pierce the surface of the gel. 4. Discard plates that have been dropped (even though the gel may not have visibly shattered). 5. Prior to conducting experiments, check the rate of gel shattering (using plates that have not been sown with seed) at different acceleration/ deceleration speeds. |

**Table S1** continued…

| Replicate size is too low. | 1. Poor germination/ seedling growth. 2. Insufficient replicate Petri plates to ensure an adequate replicate size. | 1. See above for issues relating to germination/ seedling growth. 2. Sow spare Petri plates (e.g. we sow 150 seeds across 15 Petri plates per line to guarantee >70 biological replicates for each line in each experiment). |
| --- | --- | --- |
| The adhesion properties of lines have changed. | Because roots are extremely sensitive to their environment, wild-type adhesion properties may shift between experiments.   1. Gel batches differ or environmental conditions including light, temperature or water quality fluctuated. 2. Pouring growth media too hot or incubating plates in unsuitable growth spaces can cause liquid to accumulate on the surface of the gel, disrupting adhesion, 3. A different wild-type stock was used without checking the adhesion properties of the stock. | 1. Void any experiments where excess surface water has accumulated on plates. 2. Check the growth conditions are consistent (e.g. batches of growth medium, water quality, light and temperature within the growth cabinet, etc.). 3. Ensure you run technical repeats of the assay to confirm the adhesion properties of a candidate line relative to wild-type plants are consistent. 4. Use different pooled wild-type seed stocks for a technical repeat of an experiment to ensure that the adhesion phenotype of a candidate line is consistent when a different wild-type stock is used. 5. Check and compare the adhesion properties of wild-type stocks in a single preliminary experiment. |

**Table S2** Primers used in this study for genotyping *Arabidopsis thaliana* mutants

| ***Oligo name*** | ***Oligonucleotide sequence***  ***(5’ to 3’)*** | ***Allele*** |
| --- | --- | --- |
| *jin1-9_F* | GGCGGGATTTAATCAAGAGAC | SALK_017005 |
| *jin1-9_R* | TTTGGTACAACCGCTCGTAAC |  |
| *pft1-3_F* | CGATCGAGTTGACCAAAGAAG | SALK_059316 |
| *pft1-3_R* | TTTGCATCAGGCAATATGTTG |  |
| *pdr2_F* | ATGATCCAAACAGGTGAAGAAG | SAIL_F08_811 |
| *pdr2_R* | CTATTTCTTTTGGAAACTGAGTTTGC |  |
| *abcg43-1_F* | GGGCTACAACTACAAGACGAAC | N75206^a^ |
| *abcg43-1_R* | GGGAAAAGAACAAAGAACCCAAAAG |  |
| *abcg43-2_F* | CTCGTGAAGCCAACTTGCTAG | SALK_201207 |
| *abcg43-2_R* | TCTGTAGAGTGGAAGCAACCC |  |
| *abcg43-3_F* | CCCTCAATAGAAGCTGCCTG | SALKseq_30713 |
| *abcg43-3_R* | TGTTGAGGATGATAATCGCG |  |
| SAIL_LB3 _B | TAGCATCTGAATTTCATAACCAATCTCGATACAC | SAIL_F08_811 |
| SALK_LBb1.3_B | ATTTTGCCGATTTCGGAAC | N75206^a^ SALK201207 SALKseq_30713  SALK_017005  SALK_059316 |

‘_F’ or ‘_R’ refer to the forward and reverse gene-specific primers and ‘_B’ refers to the T-DNA-specific border primer. ^a^The *abcg43-1* line was identified from a pooled set of 100 SALK T-DNA insertion lines (stock number N75206, Alonso *et al.,* 2003).
